# Is there a “Ricardian Vice”? And what is its relationship with economic policy ad“vice”?

**DOI:** 10.1007/s00191-016-0468-2

**Published:** 2016-07-09

**Authors:** Heinz D. Kurz

**Affiliations:** Graz Schumpeter Centre, University of Graz, RESOWI-Zentrum FE, Universitätsstraße 15, 8010 Graz, Austria

**Keywords:** David Ricardo, Economic policy, Innovation, Joseph Schumpeter, Ricardian Vice, Value and distribution, B12, B30, B59, D21, D78, E23

## Abstract

Schumpeter chastised Ricardo for his alleged “vice” - the so-called “Ricardian Vice” - of drawing far reaching policy conclusions from utterly simplistic models, which, moreover, were underdetermined. The paper first argues that Schumpeter saw Ricardo’s approach to the theory of value and distribution through a marginalist lens and therefore arrived at a distorted picture of the latter. Several of the criticisms he levelled at Ricardo cannot be sustained. The paper then has a closer look at Schumpeter’s pronouncements on economic policy issues and shows that in a number of respects his views did not differ that much from Ricardo’s and in some respects were remarkably similar. This concerns especially the problem of paying off the public debt, with regard to which both Ricardo after the Napoleonic Wars and Schumpeter after World War I advocated a once for all capital levy.

## Introduction

Schumpeter variously criticized Ricardo for having developed utterly simplistic models to deal with complex economic problems and, furthermore, models that are typically underdetermined. Ricardo is also said to have used these models boldly and unwarrantedly to derive wide reaching policy conclusions. With respect to these two alleged shortcomings of Ricardo’s analysis, Schumpeter spoke of the “Ricardian Vice”. Finally, he maintained that Ricardo, as did Adam Smith before him, had a wrong conception of conflicts of interest between different social groups or classes. Ricardo and Smith saw conflicts of interest, where there are none, and missed others by lumping together capitalists and entrepreneurs.

In this paper I deal with Schumpeter’s view of the role of economic theory as the foundation of economic policy. I first scrutinize Schumpeter’s criticism of the Ricardian Vice and his view of the “law of motion” of modern society. His perception of the complexity of the object of investigation makes him put forward numerous reflections and insights that are difficult to reconcile with a kind of economics that has ready-made answers to each and every problem that might possibly crop up. Schumpeter rejected what we may, for short, call “push button economics”: by pushing the right button of a model it will tell you the correct answer to whichever problem you pose. Things are not so simple, and economic models, which by their very nature are of necessity under-complex, can at best give a first, broad and preliminary orientation regarding the problem at hand. In a second step, historical, cultural, social and political specificities of the case under consideration have to be taken into account and second and third round effects of the suggested measures contemplated. This is the only way to avoid as best as possible economic policies that are ineffective or even lead to results that worsen the situation instead of improving it. This was clear to Schumpeter, but as will be seen, it was also clear to the two economists whom he criticized most: Ricardo and Keynes. Therefore, these authors typically refrain from giving definite answers to particular problems or from advocating the same kind of policy for dealing with issues that, on the surface, look similar. A one-size-fits-all approach is inappropriate, and informed reasoning has to complement economic theory and models.

The first part of this paper, consisting of Sections 1–5, scrutinizes Schumpeter’s criticisms of Ricardo. Section 2 briefly summarizes Schumpeter’s three objections above. Section 3 then shows that these are foreshadowed in the earlier literature, especially in the writings of William Stanley Jevons and Léon Walras. Section 4 argues that the first two criticisms are difficult to sustain and reflect the fact that subsequent to what has (somewhat misleadingly) been called the “marginalist revolution”, Ricardo’s analysis was seen through the lens of the new theory. This resulted in a misapprehension of the genuine significance and analytical structure of Ricardo’s “classical” approach to economic theory. Section 5 then summarizes a few important elements of Schumpeter’s theory, which sets it apart from other economic doctrines and especially the economic mainstream. The second part, consisting of Sections 6–8, turns to the problem of economic policy. Section 6 deals with Schumpeter’s writings on policy issues and tries to extract from them a general method Schumpeter applied when approaching such issues. Section 7 deals briefly with a few fields of economic policy to which Schumpeter contributed, responding to the problems of his time. Section 8 summarizes his plan to pay off the public debt Austria had accumulated during World War I and compares it to Ricardo’s plan to deal with the British public debt in the aftermath of the Napoleonic Wars. Section 9 contains some concluding remarks.

## Schumpeter on the “Ricardian Vice”

Schumpeter coined the term “Ricardian Vice” in his posthumously published *History of Economic Analysis* (1954).^1^ It was intended to chastise David Ricardo’s alleged habit of employing utterly bold assumptions into an already oversimplified representation of the economy and treating some of the magnitudes involved as givens when in fact they are unknowns. (Interestingly, he levelled the same criticism at John Maynard Keynes.) More specifically, in Schumpeter’s view, Ricardo’s “fundamental problem” was that he “wanted to solve in terms of an equation between four variables: net output equals rent plus profits plus wages” (Schumpeter 1954: 569). Ricardo, Schumpeter opined, was thus bound to treat three of the variables as constants. Closely related to this, Schumpeter also deplored Ricardo’s alleged habit of “piling a heavy load of practical conclusions upon a tenuous groundwork” (1954: 1171).^2^ Finally, according to Schumpeter, Ricardo did not merely have “an inadequate sociology, but none at all” (1954: 471). Yet had he been possessed of one, this would not have helped at all: “Given its nature, his theory would not have been improved by being caparisoned sociologically” (1954: 472). In short, to Schumpeter, Ricardo’s theory was a complete failure and beyond remedy and Ricardo’s economic proposals were devoid of any historical and social “sense”.

Interestingly, in some of his earlier writings Schumpeter was much less critical of Ricardo’s contribution. For example, in his essay on “Epochen der Dogmen- und Methodengeschichte” (Epochs in the history of economic doctrines and methods), published in 1914, he called Ricardo “the most important follower of Adam Smith”, whose *Principles of Political Economy* are said to have marked the “culminating point” of classical economics (Schumpeter 1914: 53–4). While Smith’s doctrine was “relatively superficial” (1914: 58 n. 2), Ricardo’s had both depth and coherence. However, compared to Karl Marx, Ricardo is said to have been “analytically narrow” and too inept to comprehend the “life and growth of the social body as a whole”. Whereas Smith and Marx sought to elaborate a “universal social science” (1914: 60), Ricardo was just a narrow economist.

It is particularly interesting to note that, in his 1914 essay, Schumpeter did not adopt Alfred Marshall’s (1890) assessment that modern, that is, marginalist economics, which explains relative prices and income distribution in terms of the “forces” of demand and supply, was a continuation of, and an elaboration on, the analyses of the classical economists. Classical theory and the theory of Marshall, Schumpeter (1914: 55) insisted at the time, were “connected by a loose tie” only; in substance, they were fundamentally different. Therefore, one of them could not be regarded as an early and the other as the mature version of the same theory. Unfortunately, Schumpeter later abandoned this view.

Two examples serve to illustrate what Schumpeter (1954) considered to be Ricardo’s inadmissible way of reasoning. The first is Ricardo’s famous suggestion, made in 1819, that the whole of the British national debt accumulated during the Napoleonic Wars could be paid off in a few years by means of a lump-sum tax on capital (see Ricardo 1951–1973, *Works*, vol. V: 38, 271). It would be best, he argued, if these debts were paid off as quickly as possible, *una tantum* – even at the expense of a one-time reduction in capital. After all, what would happen if the debt burden was still there when the next major emergency happened? Such a tax, Ricardo argued, would not diminish total wealth and would also not unduly hurt the propertied classes, because the capital value of the current taxes levied on them to cover interest charges and amortization was not all that different from the property tax suggested. The second example is Ricardo’s view that the burden of a tax on wages or on goods consumed by workers would not be borne by workers (e.g., Ricardo, *Works*, vol. I: 203).

Here it suffices to note that both conclusions are the logical consequence of the premise underlying Ricardo’s argument that workers are paid a given and constant real wage rate. This premise must not be interpreted as implying the factual statement that wages can never change – a sort of “iron law” of wages. However, Schumpeter (1954: 665) interpreted Ricardo’s theory of wages as implying precisely this.

Before we enter into a critical discussion of Schumpeter’s claims, it is useful to draw attention to anticipations of them in the literature. Schumpeter was very well read and may be said to have echoed them.

## Anticipations of Schumpeter’s criticism

Schumpeter was not the first to level these kinds of criticism at Ricardo. Several authors, whose writings he knew, anticipated him. It is enough to present just a few pointers to the relevant literature.

### Conflicts of interest

Schumpeter’s objections to Ricardo’s view of class conflicts were anticipated, among others, by contemporaries of Ricardo, especially Thomas Robert Malthus, commentators of various walks of life and especially representatives of the Historical Schools in England and Germany. A particularly vitriolic attack levelled at Ricardo, but strangely also at Malthus, came from the “harmonist” Henry C. Carey, who in his book *The Harmony of Interests*, published in 1851, took issue with Ricardo’s claim that the interests of landowners are opposed to those of the other classes of society: “The tendency of the whole British system of political economy is to the production of discord among men and nations. It is based upon the Ricardo and Malthusian doctrines of rent and population” (Carey 1851: preface). Ricardo and Malthus are called promulgators of “this great law of discords”.

### Lack of realism

John Maynard Keynes’s disenchantment with Ricardo’s analysis was of a different kind and, ironically, anticipated Schumpeter’s complaint about Ricardo’s inclination to draw practical conclusions from inadequate models, a complaint Schumpeter would also make against Keynes. In his essay on Malthus, Keynes called Ricardo “the abstract and a priori theorist”, a man “with his head in the clouds” (Keynes, *CW*, vol. X: 95 and 98). Ricardo’s failure to understand the *principle of effective demand* is said to have rendered his analysis not only analytically largely useless, but, much more importantly, socially harmful. “If only Malthus, instead of Ricardo, had been the parent stem from which nineteenth-century economics proceeded,” Keynes sighed, “what a much wiser and richer place the world would be to-day!” (Keynes, *CW*, vol. X: 100–101)

### Determining two unknowns from one equation

William Stanley Jevons, in *The Theory of Political Economy* (1871), objected that the classical-Ricardian theory of value and distribution was unsatisfactory not least because it started from the premise of given output levels and a given real wage rate. These magnitudes, he insisted, are to be treated as unknowns, not as data in the theory of value. Even if one was to admit that the wage rate is taken as given (reflecting the outcome of, for example, some law of population), “the doctrine is radically fallacious; it involves the attempt *to determine two unknown quantities from one equation*” (Jevons [1871] 1965: 258; emphasis added): the rate of profits and the level of output. What was badly needed, Jevons opined, was a theory of “demand” in order to close the system and render it determinate. Interestingly, exactly the same kind of incomprehension of the classical approach is present in Léon Walras’s *Elements of Pure Economics* ([1874] 1954: § 368) and then in Schumpeter.^3^

In order to assess the validity or otherwise of these objections, we have to specify briefly the characteristic features of Ricardo’s and more generally of the classical economists’ *surplus-based* approach to the theory of value and distribution.

## Ricardo’s theory of value and distribution

### Too few “equations”?

The editor of David Ricardo’s *Works and Correspondence*, Piero Sraffa, perceptively observed that the classical economists’ investigation “is concerned exclusively with such *properties of an economic system as do not depend on changes in the scale of production or in the proportions of ‘factors’*” (Sraffa 1960: v; emphasis added). In order to ascertain the general rate of profits and (relative) “natural” prices of a given economy at a given time, “No changes in output and … no changes in the proportions in which different means of production are used by an industry are considered so that no question arises as to the variation or constancy of returns” (1960: v). Hence, what the classical economists were concerned with are the (mathematical) properties of a given *system of production*, characterized by

(i) given gross output levels of the various commodities and
(ii) given methods of production to produce these outputs.

This does not mean that changes in the scale of production or in the proportions of “factors” were never analyzed by the classical economists or, worse, that they were unable to do so. It only means that they first investigated a given system of production and only in a second step investigated changes of the system (in the theory of capital accumulation and economic growth, in the theory of rent and in the treatment of the problem of the choice of technique). In addition to (i) and (ii), they typically assumed

(iii) the real wage rate to be given (or a set of real wage rates in the case of heterogeneous labor).

Could the general rate of profits and relative prices be consistently determined in terms of the data (i)-(iii)? Sraffa (1960) demonstrated that the answer to this question was yes. No other data or known variables, and especially no demand and supply functions, are needed to ascertain the unknowns. Sraffa also corroborated what became known as Ricardo’s “fundamental law of distribution”, according to which a rise in the real wage rate implies a fall in the general rate of profits, given the system of production in use.^4^

Hence the accusation put forward by Jevons, Walras and Schumpeter that Ricardo tried to determine several unknowns in terms of a single equation cannot be sustained. As early as towards the end of the nineteenth century, Knut Wicksell ([1893] 1954) and the Russian mathematical economist Vladimir K. Dmitriev ([1898] 1974: 51 et eq.) had already confirmed this. Wicksell insisted that “the way in which Ricardo develops his argument … is a model of strictly logical reasoning about a subject which seems, at first glance, to admit of so little precision”; and that “Ricardo’s theory of value is, one finds, developed with a high degree of consistency and strictness” (Wicksell [1893] 1954: 34 and 40). Dmitriev provided an early formalization of Ricardo’s approach, which was then elaborated by Ladislaus von Bortkiewicz (1906–1907). Wicksell’s book and Bortkiewicz’s essay were easily accessible, and Schumpeter can safely be assumed to have known both. Therefore it is somewhat of a riddle, why he would unswervingly retain his above criticism of Ricardo.^5^

### Ricardo – the man “with his head in the clouds”?

Was Ricardo the proverbial man “with his head in the clouds”? Obviously, this cannot be claimed with regard to the realm of banking and finance, as Keynes himself was to admit: “when it came to practical finance, the rôles of the Jewish stockbroker and the aristocratic clergyman were, as they should be, reversed” (Ricardo, *CW*, vol. X: 95). On finance, money and banking, Ricardo was knowledgeable even as regards minute technical and organizational details of the business of his time; at the Stock Exchange, he came second to none among contemporary economists. However, as Morgan (2012) has documented, he was also well informed about the progressing conditions of production in agriculture and in manufacturing. For example, there is evidence “he was familiar with the experimental farming activities of his day” (Morgan 2012: 55). He began to glimpse the emerging role of manufacturing and the machine producing industry as an “engine of growth” and the gradual industrialization of agriculture. Significantly, his chapter “On Machinery” deals with the construction and introduction of machinery in the production of “necessaries”, that is, commodities entering the production of all commodities produced in the system.^6^

An obstacle in the way of a proper understanding of Ricardo appears to be the way he reasoned, using simple conceptualizations to analyze a problem and numerical examples to illustrate it. Apparently his critics have not taken seriously his statement that “in all these calculations I have been desirous only to elucidate the principle, and it is scarcely necessary to observe, that my whole basis is assumed at random, and merely for the purpose of exemplification. The results though different in degree, would have been the same in principle. … My object has been to simplify the subject” (Ricardo, *Works*, vol. I: 121–22). Hence, while it is true that Ricardo frequently employed bold cases to clarify the principle at hand and drew attention to what he considered the most important aspects of the problem under consideration, he certainly did not wish to prevent his readers from trying out less restrictive assumptions and investigating their implications, nor did he himself abstain from doing so. Some later commentators rightly praised him for having heralded an approach in economics that requires a clear statement of the assumptions on the basis of which certain propositions are taken to be valid within a given analytical context. This is now considered an indispensable prerequisite of scientific communication. Therefore, what Schumpeter considered a vice may be considered a virtue. Further, as we shall see below, Schumpeter’s own approach, especially to economic policy problems is similar to Ricardo’s: he too typically starts from bold and highly abstract cases, but then seeks gradually to move the theoretical world within which he derives his first results towards salient features of the real world. Injecting doses of realism into the analysis makes things much more complex and often leads one into murky waters. It cannot come as a surprise then that Ricardo and Schumpeter variously arrive at similar solutions of a problem, the most important case in point being their similar attitude towards paying off huge public debts after wars (see Section 8 below).

It deserves to be stressed that Ricardo was not, as Schumpeter, amongst others, maintained, a strict follower of the Malthusian law of population and the concept of a rigid subsistence wage. Ricardo used such a concept in his debates with Malthus for the simple reason that it offered him a standpoint from which the roots of profits in the social surplus product could easily be ascertained. He did not, however, entertain the ridiculous view frequently ascribed to him that the real wage rate was a magnitude fixed forever (see Kurz 2016).

### The “natural course of things” vs. the “circular flow”

There is another aspect of Ricardo’s method of analysis that caused problems in understanding him properly. Ricardo adopted from Adam Smith the concept of “the natural course of things”, but interpreted it differently from the Scotsman. In his conceptualization, the natural course of things refers to an economy in which capital accumulates and the population grows, but in which there is no technical progress. In such a situation, rents would rise and the general rate of profits (but possibly also the real wage rate) would tend to fall. Many commentators interpreted this as meaning that the dynamic forces in the economic system were bound to lose momentum quickly, bringing the economy to a stationary state. They therefore saw in Ricardo a technological pessimist. They mistook Ricardo’s method of analysis, which involved some counterfactual reasoning, for a proposition about the actual course of things. Ricardo was perfectly clear that the actual course depends also on future “improvements” in the various sectors of the economy.

The “circular flow” in Schumpeter (1912) represents also a particular way of counterfactual reasoning, because it contemplates an economic system in which there is no change whatsoever – clearly a hypothetical situation, since “new combinations”, innovations, continually invade the actual economy and make the system grow und undergo structural transformation. In the circular flow, Schumpeter contended, there will be neither profits nor interest. These are rather said to be “both the child and the victim of development” (Schumpeter 1912: 322). Böhm-Bawerk (1913) dubbed Schumpeter’s explanation of profits therefore a “dynamic theory of capital interest.”^7^ Schumpeter’s zero-profit doctrine regarding a stationary economy was criticized from all sides: stationary conditions do not imply, by themselves, a zero rate of profits.

According to the classical authors successful “improvements” of the productive apparatus entail above normal profits for innovators (see Kurz 2008, 2010, 2015, 2016). These profits they called “extra profits” – profits above and beyond those profits yielded at the general, economy-wide level in competitive conditions. Hence, what Schumpeter called “profits” correspond to what Ricardo and the classical economists called “extra” or “supernormal profits”.

The classical authors took competition to abolish such extra profits in the course of the “gravitation” of market prices towards their “natural” levels and thus establish a uniform normal rate of profits again. Important differences notwithstanding, there is a family resemblance between this view and Schumpeter’s, who saw the centripetal force of competition to eventually annihilate profits. With the diffusion of an innovation, competitors will gradually catch up with the pioneering firm and erode its monopoly position. As a consequence, “the profits of the entrepreneur and also his entrepreneurial function as such perish in the whirlpool of the competitors that are at his heels” (1912: 286). The system moves in the direction of a new circular flow, in which the “law of cost” is reinstated again.

Due to increases in productivity that come with innovations, in any new stationary state the incomes of the broad masses are higher than in the previous one. This is Schumpeter’s version of Adam Smith’s doctrine of the unintended consequences of self-interested behavior. Behind their backs, the selfish behavior of the few causes an increase of the wealth of the many.^8^ Schumpeter thus entertains a special variant of the classical concept by conceptualizing the process of technical innovation and diffusion as a transition between circular flows: an innovation upsets the initial circular flow, its absorption by the system leads to a new one. In both, profits (and not just the extra profits of the classical economists) are nil. Schumpeter called innovations “new combinations”. The core of his analytical innovation may thus be said to consist in a reconfiguration and recombination of analytical elements he encountered in the classical economists, especially Ricardo. Hence, Schumpeter’s analysis owes a lot to an author whose groundwork he called “tenuous”. In the light of these considerations and granting the fact that Ricardo based some of his arguments on highly simplified analytical constructions, it is problematic to speak of a “Ricardian Vice”.

We now turn to Schumpeter’s analysis. We first consider its conventional side: the adoption of the neoclassical argument as it had been handed down by Léon Walras, whose general equilibrium analysis Schumpeter dubbed the “Magna Carta” of economic theory, and Alfred Marshall, whose partial equilibrium analysis he repeatedly invoked. Then we discuss briefly some “heretical” elements in his analysis, which derive partly from his readings of Marx and which put him in opposition to the Austrian and neoclassical mainstream of his time.

## Schumpeter between Walras and Marx

### The conventional Schumpeter

Much of marginalist analysis is conducted in real terms. Money is either not considered at all or taken to have no long-run effects on the real part of the economy. According to the so-called “classical dichotomy”, money is best envisaged as a veil that covers the real economy, but can be removed without much effect. (Here it suffices to observe that the adjective “classical” is misleading, because such a naive view cannot be found in Smith or Ricardo, but was adopted by some later authors.) Marginalist authors typically build up their long-period real analysis in terms of the following set of givens or known variables:

- the preferences of agents;
- the technical alternatives from which cost-minimizing producers can choose;
- the initial endowments of factors of production of the economy and their distribution among agents.

In terms of these givens, the marginalist authors then determine the prices and quantities of goods, the allocation of factors of production and income distribution.

Comparing the set of data (a)-(c) of the marginalist authors with the set (i)-(iii) of the classical economists (see Section 4) reveals important differences. In particular, in the classical authors, the distributive variables – the wage rate and the rate of profits – are not determined simultaneously and symmetrically, but consecutively: the wage rate is typically taken to be known when it comes to the determination of the general rate of profits and relative prices. In the marginalist authors, the two distributive variables are, in contrast, determined simultaneously and symmetrically by demand and supply. Clearly, to treat wages as a given in one part of classical theory is a priori no less admissible than to treat the capital endowment as given in one part of marginalist theory. Each of the two magnitudes is treated as a variable in some other part of the two theories. The only question is whether the data from which each of the two theories starts its reasoning allows one to determine in a consistent way the unknowns, especially the competitive rate of profits. As we know now, while the surplus approach of the classical economists allows such a determination, the marginalist long-period analysis faces serious difficulties (see Kurz and Salvadori 1995: chap. 14).

The asymmetric treatment of the distributive variables was unimaginable to the marginalist authors, who therefore felt entitled to accuse Ricardo of treating as a constant what is a variable, namely, the wage rate. But on what ground could one chastise Walras for having taken as given the economy’s capital endowment? To take the latter, a vector of heterogeneous commodities, as given allows one at best to determine only a short-period equilibrium characterized by differential profit rates and not *the* competitive rate, which was the magnitude sought. Yet if the capital endowment was to be given as a sum of *value* (expressed in some numeraire), as Wicksell and John Hicks suggested, the theory faces the following difficulty: according to the concept of “substitution” it advocates, cost-minimizing behavior implies that, with a rise (fall) in the real wage-to-rate of profits ratio, the labour-capital ratio falls (rises). But this is not generally true, as the capital theory debates in the 1960s and 1970s established. As Mas-Colell (1989) stressed, the relationship between the two ratios can have almost any shape whatsoever.

Schumpeter could not yet see clearly the difficulties besetting the marginalist concept of capital and its long-period analysis. Yet he seems to have sensed the possibility of unconventional relationships that question the general validity of the construction.

### The innovative and heretical Schumpeter

Schumpeter was what might be called an *enfant terrible* in the profession of economists: he praised both Léon Walras and Karl Marx for their achievements, the former as regards the realm of economic statics, the latter as regards that of economic dynamics. He saw himself firmly entrenched in mainstream economics and, at the same time, prided himself with transcending its narrow static confines. He was an intellectual entrepreneur who suggested “new combinations” of hitherto known ideas and concepts. In this section, we discuss some of Schumpeter’s most important deviations from received theory.

#### The principle of substitution: “all-dominant”? The labor market

In 1916–1917 Schumpeter published a treatise entitled “Das Grundprinzip der Verteilungstheorie” (The fundamental principle of the theory of distribution), in which he defended traditional long-period demand and supply theory against the accusation of being unable to explain the distribution of income. Large parts of the paper show a dyed-in-the wool conventional marginalist economist, emphasizing the “all-dominant principle of substitution” in production and consumption. On the basis of this principle he derives (verbally) the conventional monotonically decreasing demand schedules for factor services, that is,

$$ \partial {q}_{ij}/\partial {e}_j\le 0, $$

where *q*_*ij*_ is the amount of factor *j* needed per unit of output of commodity *i* and *e*_*j*_ is the rate of remuneration per unit of factor service *j*. But then, out of the blue, he displays his sense of “anomalies”, which undermine the doctrine he has just extolled: towards the end of his essay, in a footnote, he refers to an unconventional shape of the demand curve for labor and draws an analogy between it and the “Giffen paradox” in consumption theory. He stresses: “it could happen that consequent upon a *rise* in wages the entrepreneur finds it advantageous to forego to a larger extent quantities of *other* means of production and *increase* his demand for *labour*” (Schumpeter 1916–1917: 85–6 n.; emphases in the original).

It is not clear why there should be the purported analogy. Whilst for obvious reasons not all commodities can be Giffen goods, Schumpeter sees no problem in conceiving of the possibility of the demand for labor as a whole to be positively related to the level of wages.^9^ The negative implication of an upward sloping demand curve for labor for the theory is close at hand. With a supply curve that is assumed to be inelastic with respect to the real wage rate, the equilibrium that obtains when the two curves intersect is unstable. With free competition in the factor markets and thus perfectly flexible distributive variables, the explanation of distribution in terms of demand and supply would lead to the remarkable conclusion that deviations of the wage rate from the equilibrium level implies the complete disappearance of either wages or all other kinds of income. As Alfred Marshall stressed, unstable equilibria are of no interest. Hence an explanation of distribution would have to be sought elsewhere.

It is unclear why Schumpeter opined that the case under consideration is “of theoretical interest only”. Since he failed to state the conditions in which the case obtains, the “probability” of its occurrence cannot be decided. Interestingly, a few years earlier he had already pointed out the possibility of an unconventional investment demand curve.

#### Investment and the money rate of interest

Schumpeter (1912: chap. VI) dealt with the concept of an investment function. He implicitly rejected the marginalist view (later adopted by Keynes): investment demand need not be inversely related to the money rate of interest across the latter’s entire range of feasible levels. In an attempt to substantiate this point, he transcended the usual narrow partial equilibrium framework of the argument, based on the *ceteris paribus* assumption, by taking into account the impact of a change in the interest rate on relative prices and costs. On the one hand, a higher rate of interest implies higher prices of several goods, some of which may be needed with some of the investment projects. Whenever this is the case, the liquid capital to realize a given project will be larger, which increases the demand for credit and the volume of investment. On the other hand, a higher rate of interest implies that some projects can no longer be profitably realized, which decreases the demand for credit and the volume of investment. As regards the combined effect, nothing definite can be said a priori. There is at any rate no presumption that credit and investment demand and the money rate of interest are of necessity inversely related, as orthodox theory assumes. At least for some interval of feasible levels of the rate of interest, aggregate investment demand and the interest rate may move in the same direction.^10^

#### Savings

Adam Smith had argued that saving spurs economic development and growth: the frugal man is said to be a public benefactor, because he propels the accumulation of capital, which leads to a deeper social division of labor accompanied by a rising labor productivity. Schumpeter disputed this doctrine: for a process of development to take off, *no prior savings are needed*; it suffices to give credit to innovators. As a consequence of innovations and the higher incomes these entail, (higher) savings will obtain. The base line of Schumpeter’s argument is that investment generates savings, not the other way round. But, whereas in Keynes, another critic of the conventional view of savings, investment generates savings via an adjustment in the level of employment and the utilization of productive capacity, in Schumpeter it does so via an increase in the overall productivity of the economic system.

This becomes very clear in his bitter debate with Böhm-Bawerk, who in 1913 had frontally attacked Schumpeter’s “dynamic theory” of profits, to which Schumpeter (1913) replied, followed by a rejoinder by Böhm-Bawerk.^11^ Böhm-Bawerk argued that savings are the prime mover of economic development. Schumpeter disagreed: what matters are innovations and thus investors and not savers; bankers and capitalists and not agents abstaining from consumption; technological breakthroughs and not incremental increases in the capital stock; the “setting up of a new production function” (Schumpeter 1939: 87), and not the movement along a given one. (See also Schumpeter (1928: 381–82).)

More generally, the conventional view sees the primacy of saving over investment and that of consumers over producers. Savers decide the pace at which capital accumulates, and consumers’ preferences decide the allocation of savings in terms of actual investments in different branches of production. In this perspective, investors assume a purely vicarious role as executors of consumers’ wants and wishes. Schumpeter’s view is radically different. He stressed: “innovations in the economic system do not as a rule take place in such a way that first new wants arise spontaneously in consumers and then the productive apparatus swings round through their pressure. … It is … the producer who as a rule initiates economic change, and consumers are educated by him if necessary; they are, as it were, taught to want new things” (Schumpeter 1934: 65). In the first edition of *Theorie* he had been even more outspoken: innovators, he had insisted, “force” upon consumers new goods and consumption patterns and prompt the “hedonic majority” of the population to comply with their will. Entrepreneurs, not consumers, are the *agens* of the capitalist system. There is no undiluted consumer sovereignty in a world characterized by a stream of disruptive innovations.

If consumer preferences are influenced or even shaped by producers, they cannot be considered as entirely autonomous and as primitive data of the analysis. Schumpeter’s view of the working of the capitalist economic system questions a crucial axiom of the conventional determination of prices and produced quantities in terms of supply and demand: the independence of demand and supply functions.

Against this background, we now turn to Schumpeter’s views on economic policy. We begin with some general observations on the issue at hand, then turn to selected fields of economic policy on which Schumpeter wrote, and conclude with his a brief account of his view on paying back the public debt after the fall of the Austrian-Hungarian Empire.

## Schumpeter on economic policy in general

Schumpeter wrote numerous articles dealing with policy issues, many of them in the periodical *Der Deutsche Volkswirt. Zeitschrift für Politik und Wirtschaft* (henceforth: *DDV*), an offshoot of *Der Österreichische Volkswirt*, and in *Der Arbeitgeber* (The Employer).^12^ *DDV* was founded by Gustav Stolper in 1926. He was a close friend of Schumpeter’s; both had studied with Eugen von Böhm-Bawerk, the towering figure of the Austrian economics profession at the time, but also with Eugen von Philippovitch, an economist influenced by the younger German Historical School. Stolper supported Schumpeter whenever he could.^13^ The *DDV* swiftly became a leading intellectual magazine in Germany, but it existed only for 6 years, when Stolper, who was Jewish, in 1933 left Germany for the United States. Like the British *Economist*, *DDV* had a fairly well defined political agenda (see Rieter 1998).^14^ This revolved around the idea of a political and economic entity called “Middle Europe” – *Mitteleuropa* – and the need to defend the Weimar Republic against its enemies. The policy measures Schumpeter and Stolper advocated were all directly or indirectly at the service of these goals.^15^ Frequently, one of the two placed an article in the *DDV*, which the other then picked up and elaborated in a subsequent article, and so on. Without much of an exaggeration one might say that Stolper and Schumpeter marched separately, but stroke together. Their fundamental attitude was liberal, but they saw that historical specificities of countries had to be respected in the economic policies suggested. They were not of the opinion that there was a “one size fits all” (or *passe partout*) economic policy and therefore did not advocate plain *laissez faire*. They were opposed to what, in the above, we have called “push button economics and economic policy”. Good economic policy had to take into account as best as possible the relevant facts of the time and space, institutional and cultural characteristics, and so on. The Weimar Republic, for example, desperately needed the rebuilding and modernization of its capital stock, which had badly suffered from the war and the reparation payments imposed on Germany. Hence, Schumpeter (and Stolper) opposed all proposals that, in their view, tended to decelerate the formation of capital. This orientation towards particular goals to be achieved in given circumstances explains why Schumpeter variously came up with suggestions that could also stem, for example, from Keynes or Ricardo. In particular, he left no doubt that the transition from competitive to “trustified” capitalism necessitated a fundamental reorientation of economic policy. Schumpeter’s remarkable flexibility in respect of these matters has variously been interpreted as an expression of his opportunism – which is said to have made him tailor his argument and policy recommendations to what he expected the audience with which he dealt wished to hear.^16^ Richard Musgrave (1992: 93) spoke of Schumpeter’s “typical contrariness” – saying one thing on the one hand and even the opposite one on the other as a result of taking additional elements pertinent to the problem at hand into consideration. There is the famous joke that when ten economists are asked to come up with their views on a particular subject, 11 opinions will be presented, two coming from Mr. Keynes. Something similar could be said about Schumpeter. He was no *terrible simplificateur*, but someone who had a remarkable sense of the complexities of the social, economic and political fabric. There is frequently a *kaleidoscopic* ring to his deliberations on policy, with his argument consisting of a series of changing viewpoints, reflecting the various aspects of the problem under discussion that ought to be taken into consideration and affect the final outcome of the argument.

The deeper reason for his apparent ambiguity appears to be a fundamental contradiction between what to him is the preferred social order, which he would like to preserve, and what is the social order, which necessarily will replace the current one, given the irresistible historical forces he sees at work, transforming capitalism from within. Thus Schumpeter stressed: “Capitalism is … in so obvious a process of transformation into something else, that it is not the fact, but only the interpretation of this fact, about which it is possible to disagree.” His own interpretation of this “fact” reads:

Capitalism, whilst economically stable, and even gaining in stability [!], creates, by rationalising the human mind, a mentality and a style of life incompatible with its own fundamental conditions, motives and social institutions, and will be changed, although not by economic necessity and probably even at some sacrifice of economic welfare, into an order of things which will be merely a matter of taste and terminology to call Socialism or not. (Schumpeter 1928: 385–6)

Already 10 years earlier he had insisted that socialism’s “hour has not yet struck. … Nevertheless, the hour will come” (Schumpeter 1991: 130). In light of this, it does not come as a surprise that a mood of resignation shines through some of his writings. In fact, what sense does it make to revolt against the tide of history?

Crucial features of Schumpeter’s attitude to economic policy issues include the following. In his writings, Schumpeter initially typically sounded the horn of undiluted economic liberalism, as, for example, in his essay entitled “Dauerkrise?” (Permanent Crisis?), published in 1931 in *DDV*:

The capitalist system *as such* is not in need of regulation or planning in or outside the depression, in order to function, to be successful and to avoid breakdowns. It neither needs manipulating nor boosting, it runs by itself and engenders remarkable results, as economic history shows. The remedy for a number of screaming maladies that is logically closest at hand would not be an increase, but a decrease of interventions by central planners. Looking at the largest difficulties of today’s world economy …, one sees that in most casettrackback to industrial promotion in the past, which creates nonviable entities or blows up viable entities of nonviable dimensions.

And, with regard to the economic situation at the time, he added:

Politically unfettered and unspoilt, the capitalist mechanism would hardly bring about symptoms of such a severity and would at any rate quickly overcome them, [because what] underlies today’s economic situation is no longer the phase of depression of a normal business cycle.

Wolfgang Stolper and Christian Seidl (see Schumpeter 1985: 37) therefore stressed: “It is evident that Schumpeter, other than Keynes, conceded only a small raison d’être to economic policy makers.” While this is in fact the case, it does not mean, as we shall see, that Schumpeter saw no room for economic and more generally for social policy.

Then there are the following commandments Schumpeter advocated with respect to economic policy. *First*, any such policy has to be grounded in a given set of basic principles, which have to be established by the economist. Policy measures designed to deal with a particular problem of the day have to be derived from these principles, taking due account of the prevailing specific historical circumstances.

*Second*, since there are short- and long-term consequences of any kind of economic policy, the entire time pattern of the effects it triggers ought to be taken into consideration as best as possible. It might be the case that, whilst the short-run effects are beneficial to society, the long-run effects are detrimental to it, or vice versa. Only looking at the short run is not good enough. Alas, the short-run horizon of politicians, intent to be re-elected, makes them focus attention on the short run and care little for the long run. Political institutions may thus have a negative impact on socio-economic development.

*Third*, as with his Austrian peers and actually also Adam Smith almost a century and a half before him, Schumpeter was in favor of a judicious regulatory policy, or *Ordnungspolitik*. Such a policy involves all public measures aimed at shaping the political, economic and social framework of economic life, or the “rules of the game”, and conserving, adjusting or improving the economic order. Regulatory policy is reflected, first and foremost, in laws and social institutions (and deals with much of what is nowadays called *mechanism design*). It is concerned especially with designing

(i) a system of property rights;
(ii) a set of regulations aimed at preserving competitive conditions; and
(iii) a contract and liability law.

In Schumpeter’s view, the banking and financial sector occupied a central place in the economic system: it constituted the “head quarter” of the capitalist economy because of its role as financier of new combinations proposed by entrepreneurs. These typically do not have the means to realize their novel ideas and have to rely on credit from banks. Schumpeter, therefore, called bankers the “ephors of markets”, referring to the civil servants who in ancient Sparta were elected for a year to oversee markets. At the same time, Schumpeter was convinced that the banking and financial sector was inherently unstable and tended to deepen and prolong depressions.^17^ It was the task of economic policy – both regulatory and interventionist – to mitigate this kind of instability and thus contain its negative implications for society at large and especially for certain of its strata. Schumpeter stressed that the way in which, at the beginning of the 19th century, innovations were financed was not the only way to do so. In *Theorie* he actually expressed the conviction that the contemporary system of banking and finance was not an indispensible element of the capitalist mode of production and could be replaced with some other system, without affecting the nature of the economy and society.^18^

*Fourth*, Schumpeter was critical of process policy, or *Ablaufspolitik*. This involves a direct intervention into the economic process in terms of demand management, incomes policy, industrial policy and the like. While Schumpeter was in general skeptical as to the usefulness of such interventions – he was particularly critical of Keynesian stabilization policies – he did not, as some of his Austrian peers, reject them as a matter of principle. He saw that economic downturns, which he considered to be an integral part of business cycles and long waves of economic development, signalling the exhaustion of the potential of some “new combinations”, might request public interventions in order to fight excessive unemployment, economic distress and social misery. The process of innovation, Schumpeter was convinced, involved “creative destruction”, and, in certain conditions, the *destructive* part of the process could become unbearable for a significant part of the population and had to be fought by judicious policy measures. Especially social policy was then an appropriate tool to mitigate the necessary costs of economic progress and development and to alleviate the lot of the losers of modernization.

*Fifth*, Schumpeter insisted that economic policy ought to be steady and firm rather than erratic and volatile. This would reduce the amount of uncertainty in the process of economic decision taking and provide agents with a reliable basis to calculate costs and benefits of their actions. Due to innovations, the economic system was continuously in travail and a spasmodic economic policy would only further deteriorate the possibilities of a sober and rational calculation.

We now turn to a few policy fields out of many on which Schumpeter wrote. For a useful collection of his respective contributions, see Schumpeter (1985).^19^

## Selected fields of economic policy

### Employment policy

In a piece published in *DDV* in 1926–27 on “Unemployment”, Schumpeter deplored the lack of clarity in large parts of the population about the causes of unemployment. Yet he found even more deplorable the lack of any “sentiment of duty amongst those responsible in politics, economic life and public opinion of first to understand a phenomenon one wants to fight” (1985: 153). He defended the view put forward by Gustav Cassel in an article published in several German newspapers on 28 September 1926 and asked the rhetorical question: “Is *he* the true social policy maker, who debunks proposals that would worsen the situation and shows us the way, which *even from the standpoint of the interest of the worker* – in fact from *any* standpoint – is the right one, except from the standpoint of the politician, who would see himself checkmated like the medical doctor, all of whose patients are getting healthy?” (1985: 154)

Setting aside the problem of unemployment due to creative destruction, on which below more, Schumpeter advocated the view that, in ideal conditions, the economy would tend towards the full employment of all those seeking employment at the resulting wage rates. However, he stressed that such ideal conditions were hardly ever met and that, therefore, there is always some unemployment, which is not just search unemployment. Unemployment, he stressed, cannot exclusively be blamed on “too high” and rigid wages: social security, taxes etc. also play an important role. Most interesting perhaps, in his discussion of the sources of unemployment Schumpeter invoked what Adam Smith had called the “wretched spirit of monopoly”, that is, firms and producers being always and everywhere keen to narrow competition and establish monopolies, cartels and trusts.^20^ If successful, this entails a permanent reduction in output and employment. Persistent unemployment typically has multiple causes and not just one and, therefore, cannot be fought in terms of a singly policy instrument only.

But Schumpeter saw also the possibility that the destructive part of “creative destruction” may wipe out a large number of jobs and cause serious unemployment. If the trough of a long wave of economic development, a Kondratieff, happens to coincide with the trough of an ordinary business cycle, a Juglar, and the trough of an inventory cycle, a Kitchin, as in his view was the case in the First World Economic Crisis of the late 1920s (Schumpeter 1939: 907), then even strong measures and some demand management might be unavoidable. However, Schumpeter warned, an employment policy must not petrify the production structure and extinguish the spirit of innovation.

### Incomes policy

In his article “Lohnpolitik und Wissenschaft” (Wage policy and economic science), published in 1929 in *DDV*, he pointed out that the policy of the trade unions appears to him to aim at the maximization of the sum total of wages, which will be higher than total wages in conditions of full employment with free competition. This is so because the rise in wage rates will not be more than compensated by a fall in employment rates. For the workers who have jobs, such wage rates will be more attractive than the wage rates clearing the labor markets. Schumpeter added that even those on the dole, in case they are supported by those employed, might benefit from the higher sum total of wages. However, since the workers with jobs are not compelled to share their wages with the jobless, it is not clear how the collective improvement of all workers Schumpeter invoked could come about. He added rightly that “in a system, in which the costs of unemployment are to a large extent shifted on to other shoulders, this holds true even more so” (1985: 192).

On various occasions Schumpeter drew the attention to the *intertemporal* dimension of the dispute over the distribution of income. An increase in wages today benefits workers today and trade unions. At the same time it reduces profits and slows down the formation of capital and the diffusion of new methods of production and new goods. High or low wages (and correspondingly low or high profits) today have implications for the development and growth of the economy tomorrow and thereafter. A higher share of wages today involves a higher share of consumption and a lower share of investment today, which leads to a lower rate of capital formation and growth and thus lower levels of consumption in the future. There is an intertemporal trade-off between wages and economic growth, which, according to Schumpeter, is often ignored. Once it is taken into account, as it should be, the class conflict would be seen in a different light and lose much of its bitterness. In “Grenzen der Lohnpolitik” (Limits to wage policy), published in 1928–29, Schumpeter insisted: “*What seemed to be an opposition of class interests now turns out to be an opposition of a different kind, namely an opposition between the interests of the presence and the interests of the future*.” (1985: 194; emphasis in the original)

The implicit assumption of Schumpeter’s reasoning is, of course, that, over time, employment levels would not rise over a succession of booms and slumps compared with the lower wages scenario, and the pace at which capital accumulates would of necessity decelerate. However, if higher wages would lead to higher levels of effective demand, higher levels of employment, higher rates of capital utilization, and higher output, the trade-off would not make itself felt.

Schumpeter’s argument was tailored on the contemporaneous situation in the Weimar Republic, which, according to Schumpeter, suffered from a severe lack of capital formation. Higher wages would involve a larger share of consumption. In this way they would not only increase unemployment directly, but also indirectly via a lower rate of capital accumulation and economic growth. This would narrow the potential of wage increases in the future.

### Social partnership

Because of the interdependence of the interests of all people participating in and contributing to the economic process, Schumpeter proposed some form of industrial relations or social partnership, *Sozialpartnerschaft*. In his “Grenzen der Lohnpolitik” he anticipated an idea, which later got implemented in Austria and elsewhere. He wrote: “The day *will and must come* on which employers and workers ‘get together’ in order to discuss *uno acto wages, capital formation and tax burden*.” He concluded the article with the observation:

Nobody can help those involved, if they don’t help themselves. But they *will* help themselves and the daily progresses of the organisations of the two sides are continually getting closer towards this goal – the goal of a truly powerful machinery of negotiation, which I personally cannot imagine differently from the picture of two firmly organised monopolists of labour power und labour opportunity, which do not only settle wages, but everything that concerns them in state and society. (1985: 201; emphasis in the original)

This could pave the way to a more peaceful and rational economy and perhaps even end the class struggle.

There is a striking resemblance between Schumpeter’s idea and Rudolf Hilferding’s concept of “organized capitalism” (organisierter Kapitalismus) in *Das Finanzkapital* (1910). According to Hilferding, the upshot of the concentration and centralization of capital both in industry and in the banking sector is the emergence of two gigantic conglomerates, which use the state in order to coordinate their domestic and foreign activities. Hilferding was optimistic that, in this case, the transition to socialism was relatively easy, because the working class had just to take over the huge bureaucratic structure, and a bloody revolution could perhaps be avoided. Schumpeter’s concept may be considered the soft version of Hilferding’s organized capitalism.

In concluding, there is one aspect of Schumpeter’s view that ought to be stressed. Schumpeter variously emphasized that entrepreneurship is not limited to the economic realm: the dichotomy of “energetic” and “innovative” people, on the one hand, and “hedonic” and “rationalistic” ones, on the other, is also to be found in the world of culture and the arts, the sciences, and, last but not least, in politics and public administration; see, for example, Schumpeter (1912: 544–47). It matters who governs a country, to which kind of incentives the public administration is exposed, and so on. As Mazzucato (2013) showed, it is myth that the private sector is always productive and innovative, whereas the public sector is always unproductive and wasteful.

## Paying off the public debt

In 1918 Schumpeter published an essay entitled “Die Krise des Steuerstaates” (Schumpeter 1918, 1991) in which he discussed critically Rudolf Goldscheid’s views contained in the latter’s widely read book *Staatssozialismus oder Staatskapitalismus*. The two contributions started a new field of inquiry in the social sciences: fiscal sociology. Schumpeter’s essay was published a few months before he was appointed by Otto Bauer upon a recommendation of Rudolf Hilferding to the position of Finance Minister in the coalition government of Social democrats and Christian democrats of the newly formed Austrian Republic (so-called Government Renner II). The essay documents Schumpeter’s wide range of interests and knowledge and deals with the problem of the limits of the tax state in its broader social and political context. The question was: To what extent was the tax state of parliamentary democracies able to sustain expanding budgets due to wars, economic crises, social needs and wants etc. Goldscheid had advanced the view that the tax state was bound to run into difficulties because capitalism was crisis-prone and economically declining and thus could not cope with the problem. A fundamental change in the socio-economic order was needed – a transformation of state capitalism in state socialism.

While Schumpeter agreed with parts of Goldscheid’s analysis, he did not agree with his conclusions. It was not the malfunctioning of the capitalist economy that threatened the tax state. It was rather the expansion of fiscal needs due to expanding social programs etc. that threatened the capitalist economy. In particular, Schumpeter was convinced that Austria’s major post-war problem – its huge public debt – could be solved within the given socio-economic order. Space constraints prevent me from entering into a discussion of all the fascinating details and multifarious themes Schumpeter dealt with in the essay. A few remarks must suffice.

Schumpeter agreed with Goldscheid that the fiscal potential of the tax state is limited: “The tax must not demand from the people so much that they lose financial interest in production” (Schumpeter 1991: 112) – it must not destroy the incentive to improve their situation, work hard and be creative. While taxing monopoly rents and the rents of land can be expected to have little implications for the incentive to produce, things are different with respect to entrepreneurial profits: taxing them might slow down the process of innovation and paralyze “the driving forces of the economy” (1991: 115). Schumpeter therefore was against high taxes on capital and wealth. However, exceptional conditions might require exceptional means.

In order to pay back the huge public debt in post-war Austria, Schumpeter advocated a once and for all capital levy. Such a levy was said to be both feasible and compatible with the prevailing form of socio-economic organization. Since the cost of the war in real terms has already been borne, the capital levy only skims off excessive liquidity and does not affect the formation of new capital. Hence, in Schumpeter’s view, there was no crisis of the tax state that threatened its existence. The problem was solvable; it required a political class capable of putting the proposed scheme in action.

In his capacity as Finance Minister, a position he held for only 7 months, Schumpeter worked out a plan along the lines expounded in his 1918 essay. According to it, the major part of the capital levy would have to be paid over 3 years and the proceeds deactivated in order to curb excess money circulation, fight inflationary tendencies and return to a sound currency. Musgrave (1992: 93) summarized the rest of the plan as follows: “The remainder would be paid by pledging a contribution from private capital income, designed to attract foreign capital, thereby underwriting the dependability of future debt service.” In addition, Schumpeter suggested to complement the plan by a tax reform, with tax rates on middle income ranges somewhat higher than before, a smaller top rate of income tax of just 33 %, and an increase of indirect taxes on upmarket consumption goods. In this way, paying off the public debt was taken not to frustrate the badly needed capital formation.

The social democratic ministers in the cabinet, led by Otto Bauer, did not approve of Schumpeter’s plan. They sought to tackle the debt and other problems in terms of the gradual socialization of substantial parts of the Austrian economy. Schumpeter’s excursion into politics was not crowned by success.

## Concluding remarks

Our argument has brought us back to its beginning. Ironically, Schumpeter’s capital levy plan was not so different from Ricardo’s proposal to pay off England’s public debt after the Napoleonic Wars, which, as we have seen in Section 2 above, Schumpeter chastised as an expression of the “Ricardian Vice”. Why did Schumpeter not pass the same judgement on his own proposal? The following considerations may contribute to explaining why.

First, as we have heard, Schumpeter had a very low opinion of Ricardo’s theory and a very high opinion of Walras’s. As we have also heard, he was convinced that economic policy recommendations had to be solidly grounded in a set of fundamental principles derived from economic theory. Echoing Walras’s view,^21^ Schumpeter felt that Ricardo’s theory was either wrong or simply an ad hoc construction designed to serve some preconceived political stance. How then could Ricardo’s policy recommendations be worth anything? Possessed of what Schumpeter considered to be a much superior theory, composed of two parts, Walras’s for stationary conditions and his own for dynamic ones, how could he not be convinced that his own proposals were supported by the most advanced knowledge in economics? In cases in which he and Ricardo happened to arrive at similar policy conclusions, such as in the debt question, it was clear that Ricardo must have arrived there for the wrong reasons purely by chance, whereas he, Schumpeter, arrived there for the best ones available at the time.

Second, according to Schumpeter, a further argument spoke in favor of the solidity of his own policy recommendations. As we have heard, he prided himself, at least indirectly, with being possessed of an adequate sociology, whereas Ricardo had none at all. By this he meant that his economic deliberations were embedded in a rich sociological perspective on the problem at hand that took due account of the political, cultural and institutional circumstances shaping the possibilities and confines of economic and social policy measures. This may very well be true, but it is certainly an exaggeration to claim that Ricardo had no sense whatsoever of the wider sociological aspects of the problems which with he dealt in terms of his theoretical models. His activities in parliament demonstrate vividly his broad socio-economic, political and cultural concerns. Many of the documents bearing testimony to this Schumpeter could not know, because they had not yet been published. Furthermore, the socio-economic realities confronting the two authors differed in important respects – economically, politically and socially. In particular, Ricardo did not yet experience industrial cycles, which occurred only after the capitalistically organized industrial sector had assumed a certain extension and profundity. This was the case at around the time when Ricardo passed away.^22^

Schumpeter, as we have seen, was overly optimistic as regards the consistency of Walras’ theory and grossly underrated that of Ricardo’s. His complaint that Ricardo had the tendency of “piling a heavy load of practical conclusions upon a tenuous groundwork” is to be amended. First of all, Ricardo’s “groundwork”, while not without shortcomings and gaps, can hardly be called “tenuous”, having arguably been the best available at the time. The practical conclusions Ricardo drew from it were thus also not as ill-founded, as his critics, including Schumpeter, maintained. When his adversaries in Parliament denounced Ricardo as someone who has “just descended from some other planet” (Ricardo, *Works*, vol. V: 85), this was not because he lacked practical knowledge and analytical judgement on the matters under consideration. It was rather because his adversaries understood well that his proposals were not only well argued, but harmful to their economic interests. Ricardo, concerned with what he felt was in the interest of the body politic as a whole, opted for a lump-sum tax on capital (including landed property and financial means) to pay back the post-Napoleonic British debt in full recognition of the fact that he would be amongst those affected most by such a tax.^23^

Economic advisers to politicians often deplore the fact that their counterparts are not willing to listen long and carefully, abhor sophisticated arguments, expect to be given simple and straightforward answers that do not deviate too much from their preconceived opinions, are possessed of a short time horizon reflecting the election cycle and frequently do not care much about what the advisors recommended. The principle that, according to Einstein, ought to apply to scientific work – “Everything must be made as simple as possible, but not simpler” – typically is not applied in policy counselling: too simple is hardly ever simple enough. One can thus only wonder why “economic policy advice” contains the noun “vice”.

## Footnotes

1 However, he used the concept, but not the term, already much earlier, for example in a review of Keynes’s *General Theory* and in his obituary of Keynes. See also Kurz (2009, 2015).

2 Charges of this kind are not uncommon in economics. See, for example, Leontief (1937), who accused Keynes of “implicit theorizing”.

3 It is also encountered in more recent times; see, for example, Arrow (1991).

4 For a thorough discussion of the classical theory of value and distribution, allowing for fixed capital, joint production and scarce natural resources, see Kurz and Salvadori (1995). For a comparison of Schumpeter and the classical economists on profits and technical change, see Kurz (2008).

5 There is evidence that Schumpeter was analytically not always up to the task he put to himself. For example, he extolled Walras’s general equilibrium theory, but apparently was not aware of any of its shortcomings, some of which Bortkiewicz had pointed out in review articles of Walras’s works he published in the 1890s; see Gehrke and Kurz (2016). In the *History of Economic Analysis*, Schumpeter (1954: 851) expressed a fair amount of incomprehension of Bortkiewicz’s remarkable analytical achievements by calling him a “comma hunter”, who “had no eye for the wider aspects and deeper meanings of a theoretical model.” My guess therefore is that Schumpeter trusted Walras’s judgement more than Wicksell’s or Bortkiewicz’s and, as a consequence, simply put the objections to one side.

6 “Luxuries”, on the other hand, do not satisfy the above criterion. To Ricardo, the distinction was of paramount importance, because the general rate of profits depended only on the conditions of production of necessaries, not on those of luxuries.

7 On the controversy between Schumpeter and Böhm-Bawerk, see Kurz (2012: 888–892 and 2013a).

8 It must be stressed, however, that Smith was *not* of the opinion that nothing but self-regard or even selfishness was needed to yield socially beneficial outcomes. Institutions and a regulatory framework rather had to prevent selfish behaviour from developing its dark and destructive sides. See on this Kurz (2016).

9 It is interesting to note that in a reaction to the debate about the so-called reswitching of techniques, Joseph Stiglitz (1974) also drew an analogy to the Giffen paradox.

10 The case is illustrated in Kurz (2012: 895–6). Schumpeter appears to have assumed that the ranking of the investment projects does not change with a change in the interest rate. However, it is well possible that the projects cannot be ordered monotonically irrespective of the level of the interest rate. Up until recently the demonstration that certain relationships need not exhibit the usual properties postulated by marginalist theory started from the assumption of a positive rate of interest (or profits) and thus relied on compound interest effects. However, Opocher and Steedman (2015) have shown that non-conventional input demand-input price relationships may also result in the case of a zero rate of interest.

11 For a summary account of the debate, see Kurz and Sturn (2012: 61–71).

12 It ought to be mentioned that after his debacle as a banker, he had to pay back a large debt, which forced him to accept various jobs as speaker and writer in order to earn some extra money on the side.

13 Without Stolper’s and August Spiethoff’s support, it is doubtful that Schumpeter would have been appointed to a chair in economics at the University of Bonn in 1926; see Kurz and Sturn (2012) and Kurz (2013b).

14 It deserves to be noted that when Stolper invited Schumpeter to regularly contribute articles to the *DDV*, he wrote to him: “I have in mind that you do something similar for *Der Deutsche Volkswirt*, what Keynes does for *The Nation*” (see Schumpeter 1985: 34).

15 In his review article of Keynes’s *General Theory*, Schumpeter accused Keynes of the Ricardian Vice and contended: “everywhere he really pleads for a definite policy, and on every page the ghost of that policy looks over the shoulder of the analyst, frames his assumptions, guides his pen” (Schumpeter 1936: 792). In the light of what has been said above, one can only wonder whether Schumpeter never fell victim to the vice. After all, as he stressed, man is guided by “visions” and even “ideologies” and these may triumph over what economic theory allows us to say.

16 According to Stolper and Seidl (see Schumpeter 1985: 7), Schumpeter in the turmoil after World War I was keen to prevent the worst from happening and to rescue what could be rescued. This made him jump at a chance whenever he saw one, which explains his fickleness that has been interpreted as opportunism.

17 Hyman Minsky, author of the financial instability hypothesis, was a student of Schumpeter’s with a strong Keynesian leaning.

18 It deserves to be mentioned that Schumpeter propagated the concept of venture finance and was an unsuccessful venture investor himself during a brief period at the beginning of the 1920s; see Peneder and Resch (2015).

19 In the following, all page references relate to the reprint of Schumpeter’s papers (1985).

20 Here we do not refer to the temporary monopolies, which, according to Schumpeter, are a necessary part of the process of innovation. We rather refer to the re-organization of markets, the establishment of lasting monopolies and so on, which Schumpeter, interestingly enough, counts amongst “new combinations” just like new goods and new methods of production.

21 See Kurz and Salvadori (2002), who discuss in detail Walras’s criticism of Ricardo’s theory of value and distribution and show that Walras failed to see that the classical approach was fundamentally different from his own demand-and-supply approach. He treated Ricardo’s theory as if it was just an early and rude version of his own elaborate neoclassical theory. Unfortunately, Schumpeter fell victim to Walras’s misconception.

22 In chapter XIX, “On sudden changes in the channels of trade” of the *Principles* (Ricardo, *Works*, vol. I: 263–72), Ricardo was concerned mainly with issues such as the distress in some industries caused by the removal of capital from it to some other industry and especially the transition from a peace to a war economy and vice versa during the time of the Napoleonic wars. A succession of good and bad harvests in agriculture could, of course, cause ups and downs in the economy, the source of which, however, was exogenous and not endogenous, the case in which Schumpeter was interested.

23 Schumpeter’s wealth and property at the time he held the office of finance minister was modest; the tax he proposed would not have affected him much.
